# Prevention of mother-to-child transmission of HIV in Kenya: challenges to implementation

**DOI:** 10.1186/1472-6963-14-S1-S10

**Published:** 2014-05-12

**Authors:** Elsabé du Plessis, Souradet Y Shaw, Mary Gichuhi, Larry Gelmon, Bensen B Estambale, Richard Lester, Joshua Kimani, Lisa S Avery

**Affiliations:** 1 Centre for Global Public Health, Department of Community Health Sciences, Faculty of Medicine, University of Manitoba, Winnipeg, Canada; 2 Department of Medical Microbiology, Faculty of Medicine, University of Manitoba, Winnipeg, Canada; 3 Medical Microbiology, University of Nairobi, Nairobi, Kenya; 4 Division of Infectious Diseases, Department of Medicine, University of British Colombia, Vancouver Canada

**Keywords:** PMTCT, vertical transmission of HIV, guideline adherence, Kenya, PTME, transmission verticale du VIH, respect des lignes directrices, Kenya

## Abstract

**Background:**

The prevention of mother-to-child transmission of human immunodeficiency virus (HIV) is lauded as one of the more successful HIV prevention measures. However, despite some gains in the prevention of mother-to-child transmission of HIV (PMTCT) in sub-Saharan Africa, mother-to-child transmission rates are still high. In Kenya, mother-to-child transmission is considered one of the greatest health challenges and scaling up PMTCT services is crucial to its elimination by 2015. However, guideline implementation faces barriers that challenge scale-up of services. The objective of this paper is to identify barriers to PMTCT implementation in the context of a randomized control trial on the use of structured mobile phone messages in PMTCT.

**Methods:**

The preliminary analysis presented here is based on survey data collected during enrolment in PMTCT services at one of two health facilities in Nairobi, Kenya, with overall number of antenatal care (ANC) visits determined from 48 hour follow up data.

**Results:**

Data was collected for 503 women. Despite significant differences in the type of facility and sample characteristics between sites, all women presented to care at 20 weeks gestation or later and 88.8% attended less than four ANC visits. PMTCT counselling at first visit had high coverage (86%), however less than a third of women (31.34%) reported receiving contraception counselling. Although 60.8% of women had reportedly disclosed their status to their partners, only 40% were aware of their partner’s status. Very few women had been tested for TB (10%) or received single dose nevirapine during their first antenatal care appointment (20%).

**Conclusion:**

Revised PMTCT guidelines aim to reduce the inequity between PMTCT services in high and low resource settings in efforts to eliminate mother-to-child transmission. However, guideline implementation in low resource settings continues to be confronted with challenges related to late presentation, less than four ANC visits, low screening rates for opportunistic infections, and limited contraception counselling. These challenges are further complicated by lack of disclosure to partners. Effective scale up and implementation of PMTCT services requires that such ongoing program challenges be identified and appropriately addressed within the local context.

## Abstract (fr)

**Résumé:**

**Contexte**

La prévention de la transmission du virus de l’immunodéficience humaine (VIH) de la mère à l’enfant est reconnue comme l’une des méthodes de prévention du VIH les plus efficaces. Cependant, bien que l’on ait réalisé certains gains au chapitre de la prévention de la transmission du VIH de la mère à l’enfant (PTME) en Afrique subsaharienne, les taux de transmission de la mère à l’enfant demeurent élevés. Au Kenya, la transmission de la mère à l’enfant est considérée comme l’un des plus grands problèmes de santé, et il est crucial de faire passer à grande échelle les services de PTME si l’on veut arriver à éliminer la transmission d’ici 2015. Toutefois, la mise en oeuvre de lignes directrices est entravée par des obstacles qui freinent le passage à grande échelle des services. L’objectif du présent article est de présenter les facteurs qui font obstacle à la mise en oeuvre de la PTME dans le contexte d’un essai comparatif avec répartition aléatoire sur l’utilisation d’un système de messages uniformisés pour la PTME.

L’analyse préliminaire présentée ici s’appuie sur des données d’enquête recueillies durant l’inscription aux services de PTME à l’un de deux établissements de santé de Nairobi, au Kenya, où le nombre de visites en soins prénataux. Les données de suivi recueillies dans les 48 heures ont permis de déterminer le nombre de visites en soins prénataux.

**Résultats**

Les données recueillies portaient sur 503 femmes. Si les deux établissements étaient très différents et que les caractéristiques de l’échantillon variaient beaucoup d’un établissement à l’autre, toutes les femmes se sont présentées à 20 semaines de gestation ou plus tard, et 88,8 % d’entre elles se sont présentées à moins de quatre visites en soins prénataux. Bien que 86 % des femmes aient reçu des services de counselling en PTME, moins du tiers (31,4 %) ont indiqué avoir reçu des conseils sur la contraception. De plus, tandis que 60,8 % des femmes ont dit avoir révélé leur statut sérologique à leur partenaire, seulement 40 % d’entre elles connaissaient celui de leur partenaire. Très peu de femmes avaient subi un test de dépistage de la tuberculose (10 %) ou reçu une dose unique de névirapine à l’occasion de leur premier rendez-vous en soins prénataux (20 %).

Les lignes directrices en matière de PTME visent à réduire l’écart en matière de services de PTME entre les milieux favorisés et les milieux démunis afin d’éliminer la transmission de la mère à l’enfant. Cependant, la mise en oeuvre de ces lignes directrices dans les milieux défavorisés continue d’être entravée par des obstacles reliés aux premiers rendez-vous tardifs, au nombre limité de visites (moins de quatre), aux faibles taux de dépistage des infections opportunistes et au manque de services de counselling en contraception. Le fait que certaines femmes ignorent le statut sérologique de leur partenaire ne fait qu’aggraver ces problèmes. Le passage à grande échelle et la mise en oeuvre des services de PTME exigent que ces problèmes récurrents soient ciblés et abordés adéquatement en fonction du contexte local.

## Background

The prevention of mother-to-child transmission of human immunodeficiency virus (HIV) is lauded as one of the more successful HIV prevention strategies, with substantial reductions in the number of HIV positive children born to HIV positive women reported. Globally, it is estimated that 390 000 new paediatric infections were recorded in 2010, 15% less than reported in 2001 [1]. These reductions, however, seem concentrated in the developed world where there is greater coverage of prevention of mother-to-child transmission of HIV (PMTCT) services and antiretroviral therapy (ART) [2]. Despite some gains in PMTCT in sub-Saharan Africa (SSA), rates of mother-to-child transmission are still high and 90% of paediatric infections occur here, highlighting the growing inequalities in global health [1,3].

In Kenya, the mother-to-child transmission of HIV is considered to be one of the greatest health challenges with an estimated 37 000 to 40 000 infants infected annually [3]. Scaling up PMTCT services in the country is crucial for Kenya to eliminate mother-to-child transmission by 2015, as recently called for by the Joint United Nations Programme on HIV/AIDS. The implementation of current PMTCT guidelines, however, faces certain barriers that would also challenge scale-up. A recent review of the implementation of Kenya’s PMTCT guidelines found that although certain aspects of PMTCT services, such as counselling and ART, had achieved reasonable coverage, other aspects such as disease staging had more limited coverage [4]. Although the development of national guidelines is one strategy to increase the effectiveness of PMTCT services offered by healthcare providers [3], inconsistent implementation of guidelines in low income countries could be a hindrance to PMTCT services.

### PMTCT guidelines in Kenya: 2012

In 2012, Kenya published revised PMTCT guidelines based on WHO guidelines published in 2010 [3]. Based on the four-pronged approach promoted by the WHO, it focuses on primary prevention of HIV infection in women, prevention of unintended pregnancies, reducing transmission during pregnancy, labour and breastfeeding, and providing support to HIV-positive women and their families. As such, the Kenyan guidelines encourage four or more antenatal care (ANC) visits with an essential package of services that includes (but is not limited to) counselling, medical history and examination, nutritional assessment, testing for opportunistic infections including tuberculosis (TB), positive prevention counselling (including disclosure, condom use, psychosocial support for families), and an effective contraception plan. The revised guidelines have a much greater focus on pharmaceutical prophylaxis than previous guidelines and promote earlier initiation of therapy for all pregnant women. Women who are eligible to receive ART (CD4 cell count of 350 or below with WHO clinical stage of I or II, or WHO clinical stage III or IV, regardless of CD4 cell count) should be started on highly active antiretroviral therapy (HAART) regardless of gestational age. Women not eligible for HAART should be started on combination antiretroviral (ARV) prophylaxis at 14 weeks, or shortly thereafter and receive a combination of AZT, 3TC, and NVP at the onset of labour. The Kenyan guidelines include Option A (single dose nevaripine in labour) although option B is encouraged in settings with the capacity to monitor women receiving triple therapy. This could also be continued through the woman’s life without interruption, known as option B PLUS. Infants should receive single-dose nevirapine (sdNVP) within 72 hours of delivery, followed by 3TC and AZT. Infants should receive nevirapine six weeks following birth if the mother was on HAART before pregnancy or is not breastfeeding. If the mother is breastfeeding, the infant should receive nevirapine from birth to one week following the last exposure to breast milk. The guidelines also instruct that, at the first ANC visit, all HIV infected pregnant women should be given sdNVP for themselves (to be taken at the onset of labour) and for the infants-“to be administered soon after birth” [3].

Kenya’s revised guidelines adhere closely to the best evidence for PMTCT as captured in the WHO guidelines and could contribute to the elimination of mother-to-child transmission by 2015, if implemented across the country. Barriers to implementation, however, could lead to inconsistent implementation and reduce the guidelines’ impact. This paper examines adherence to aspects of Kenya’s PMTCT guidelines, including number of antenatal care visits, counselling (including family planning and disclosure), and TB testing, based on data collected during a study of mobile health (mHealth) in PMTCT service delivery.

## Methods

### Study design and sampling

These results were obtained in the context of a randomized control trial (RCT) on the use of structured mobile phone messages in PMTCT. The goals of the study were to improve linkage to ANC, provide reminders to take PMTCT medications, and improve post-natal support and follow-up – even when mothers deliver elsewhere. In addition to benefits directly related to PMTCT, the study also aimed to demonstrate that mobile phone technology can be used as an effective tool for strengthening health information systems at a facility level by collecting better information, and thereby advance local health systems development.

Women were recruited from the PMTCT clinic at Pumwani Maternity Hospital (PMH) and the ANC/PMTCT clinic at Baba Dogo Health Centre (BDHC) in Nairobi, Kenya and enrolled in the study during their first ANC visit, if they met the inclusion criteria. They were considered eligible if they were HIV positive, pregnant with a singleton pregnancy, had never had a preterm birth, were residing within 15 km of either study facility, and would be residing there for at least six months post-delivery, literate in Kiswahili or English, were willing to be contacted for follow up, and had regular access to a cell phone – either their own or that of their partner or a family member. Disclosure of HIV status to their partner was initially included as criteria but was eliminated at the request of key stakeholders who thought this would not be representative of women receiving PMTCT services in Kenya. Women who were considered eligible were approached by study nurses –not affiliated with either facility- who explained the objectives and procedures of the study and asked if they wanted to participate (one nurse was hired at each facility). Nurses had experience working in maternal health as well as previous experience working on research projects. If women agreed to participate, informed consent was obtained before conducting the baseline survey or collecting samples.

### Data collection and analysis

Survey data was collected at enrollment, during routine ANC visits, and 48 hours and 6 weeks post-delivery. At enrollment, women completed a brief questionnaire collecting data on socio-demographic characteristics, cell phone history, HIV status history, previous pregnancy, current pregnancy, gender based violence, TB testing and care, and PMTCT knowledge. Subsequent data collection focused on ARV history, pregnancy complications, delivery and postpartum data, and perceptions of the mobile phone intervention (intervention clients only).

The preliminary analysis presented here focuses on the survey data collected at enrolment with the exception of the number of ANC visits which was determined from data collected at the 48 hour-follow up. We compared variables of interest across the two hospital sites as we were interested in determining whether or not there were differences between sites, given the differences in populations served and clinical practice. Our assumption was that while there may be differences by site, there may also be broader structural barriers we could identify at both sites. Data was analysed using Stata 11 (College Station, TX) and descriptive analyses included comparisons between women recruited from the different hospitals. Chi-square tests of association were used to compare variables of interest across the two hospital sites.

### Ethical considerations

Ethics approval was obtained from the University of Manitoba’s Health Research Ethics Board (H2009-351) as well as the Kenyatta National Hospital/University of Nairobi - Ethics & Research Committee (P273/09/2009). Women provided signed informed consent to participate in the study.

## Results

Data was available for 503 women (325 from PMH and 178 from BDHC), ranging in age from 18 to 49. Table 1 includes a description of the sample characteristics broken down by site. The average age was 29.4 and the majority of women were married (78.5%). There were significant differences between the groups recruited from the different facilities: women accessing services at BDHC were younger, (74.1% were under 30 years of age, as opposed to 43.1% at PMH), predominantly Luo (70.8%), and of lower socioeconomic status. They were also more likely to have moved to the area within the past two years.

**Table 1:** Socio-demographic characteristics of sample (N;%)

	Variables	Pumwani (N=325)	Baba Dogo (N=178)	Total (N=503)	p
Age group	18-21 22-24 25-29 30-34 35-39 40+	9 (2.8) 32 (9.8) 101 (31.1) 98 (30.2) 62 (19.1) 23 (7.1)	20 (11.2) 46 (25.8) 66 (37.1) 32 (18.0) 11 (6.2) 3 (1.7)	29 (5.8) 78 (15.5) 167 (33.2) 130 (25.8) 73 (14.5) 26 (5.2)	0.000
	
	Total	325 (100.0)	178 (100.0)	503 (100.0)	

Ethnicity	Kikuyu Luo Kamba Luhya Kisii/Kalenjin/Masai Other	101 (31.1) 97 (29.8) 47 (14.5) 36 (11.1) 15 (4.6) 29 (8.9)	13 (7.3) 126 (70.8) 13 (7.3) 21 (11.8) 4 (2.2) 1 (0.6)	114 (22.7) 223 (44.3) 60 (11.9) 57 (11.3) 19 (3.8) 30 (6.0)	0.000
	
	Total	325 (100.0)	178 (100.0)	503 (100.0)	

Education	Primary Secondary Post-Secondary	154 (47.4) 121 (37.2) 50 (15.4)	113 (63.5) 55 (30.9) 10 (5.6)	267 (53.1) 176 (35.0) 60 (11.9)	0.000
	
	Total	325 (100.0)	178 (100.0)	503 (100.0)	

Marital status	Single Married Divorced/Widowed/Separated	44 (13.5) 250 (76.9) 31 (9.5)	23 (12.9) 145 (81.5) 10 (5.6)	67 (13.3) 395 (78.5) 41 (8.2)	0.286
	
	Total	325 (100.0)	178 (100.0)	503 (100.0)	

Husband/partner’s profession	Civil service Self-employed Missing Other	20 (6.2) 149 (45.8) 68 (20.9) 88 (27.1)	7 (3.9) 27 (15.2) 33 (18.5) 111 (62.4)	27 (5.4) 176 (35.0) 101 (20.1) 199 (39.6)	0.000
	
	Total	325 (100.0)	178 (100.0)	503 (100.0)	

Respondent’s profession	Civil service Self-employed Caregiver Other	9 (2.8) 113 (34.8) 152 (46.8) 51 (15.7)	4 (2.2) 38 (21.3) 103 (57.9) 33 (18.5)	13 (2.6) 151 (30.0) 255 (50.7) 84 (16.7)	0.016
	
	Total	325 (100.0)	178 (100.0)	503 (100.0)	

Monthly income	< 5,000 KSH 5,000 - <10,000 KSH 10,000 - <15,000 KSH 15,000+ KSH	149 (45.8) 85 (26.2) 38 (11.7) 53 (16.3)	76 (42.7) 63 (35.4) 23 (12.9) 16 (9.0)	225 (44.7) 148 (29.4) 61 (12.1) 69 (13.7)	0.042
	
	Total	325 (100.0)	178 (100.0)	503 (100.0)	

How long lived in area	<1 Year 1 - <2 Years 2 - < Years 3+ Years	55 (16.9) 30 (9.2) 33 (10.2) 207 (63.7)	54 (30.3) 21 (11.8) 21 (11.8) 82 (46.1)	109 (21.7) 51 (10.1) 54 (10.7) 289 (57.5)	0.001
	
	Total	325 (100.0)	178 (100.0)	503 (100.0)	

### HIV and treatment history

More than half the women reported contracting HIV more than a year before the pregnancy and 62.6% were receiving ART, with the majority of infected women reportedly initiating therapy within the previous year (Table 2). The most common reason given for initiating therapy was low immunity. There were significant differences between women recruited from different sites in terms of HIV status and access to ART as women accessing services at PMH reported being HIV-positive for longer than women at BDHC and were more likely to be receiving ART, possibly due to the older average age (Table 2).

**Table 2:** HIV and treatment history (N;%)

	Variables	Pumwani (N=325)	Baba Dogo (N=178)	Total (N=503)	p
Length had HIV	<1 Year 1 - <2 Years 2 - <3 Years 3+ Years	138 (42.5) 35 (10.8) 37 (11.4) 115 (35.4)	97 (54.5) 30 (16.9) 15 (8.4) 36 (20.2)	235 (46.7) 65 (12.9) 52 (10.3) 151 (30.0)	0.001
	
	Total	325 (100.0)	178 (100.0)	503 (100.0)	

Where received HIV diagnosis	ANC VCT Mobile clinic Other	176 (54.2) 77 (23.7) 13 (4.0) 59 (18.2)	104 (58.4) 62 (34.8) 2 (1.1) 10 (5.6)	280 (55.7) 139 (27.6) 15 (3.0) 69 (13.7)	0.000
	
	Total	325 (100.0)	178 (100.0)	503 (100.0)	

On ARV	Not on ARV On ARV	97 (29.8) 228 (70.2)	91 (51.1) 87 (48.9)	188 (37.4) 315 (62.6)	0.000
	
	Total	325 (100.0)	178 (100.0)	503 (100.0)	

Why are you on ARV	Low immunity Pregnancy Other	136 (60.2) 86 (38.1) 4 (1.8)	63 (72.4) 23 (26.4) 1 (1.1)	199 (63.6) 109 (34.8) 5 (1.6)	0.131
	
	Total	226 (100.0)	87 (100.0)	313 (100.0)	

How long on ARV	<1 Year 1 - <2 Years 2 - <3 Years 3+ Years	126 (55.5) 30 (13.2) 23 (10.1) 48 (21.1)	42 (48.3) 17 (19.5) 10 (11.5) 18 (20.7)	168 (53.5) 47 (15.0) 33 (10.5) 66 (21.0)	0.491
	
	Total	227 (100.0)	87 (100.0)	314 (100.0)	

### Antenatal care

The majority of women presented for their first ANC visit between 21 and 28 weeks gestation (table 3) with only 15.3% of women attending their first ANC visit at 20 weeks gestation or less. The vast majority of women attended less than four ANC visits (88.8%).

**Table 3:** PMTCT characteristics (N;%)

	Variables	Pumwani (N=325)	Baba Dogo (N=178)	Total (N=503)	p
Number of ANC visits, current pregnancy	<4 4+	272 (84.7) 49 (15.3)	171 (96.1) 7 (3.9)	443 (88.8) 56 (11.2)	0.000
	
	Total	321 (100.0)	178 (100.0)	499 (100.0)	

Gestational age at first ANC visit	≤20 weeks 21-28 weeks >28 weeks	38 (11.7) 155 (47.7) 53 (40.6)	21 (11.8) 104 (58.4) 53 (29.8)	59 (11.7) 259 (51.5) 185 (36.8)	0.044
	
	Total	325 (100.0)	178 (100.0)	503 (100.0)	

Told about PMTCT, 1^st^ ANC visit	No Yes	49 (15.2) 274 (84.8)	21 (11.8) 157 (88.2)	70 (14.0) 431 (86.0)	0.297
	
	Total	321 (100.0)	178 (100.0)	501 (100.0)	

Given nevirapine, 1^st^ ANC visit	No Yes	194 (77.6) 56 (22.4)	138 (84.1) 26 (15.9)	332 (80.2) 82 (19.8)	0.102
	
	Total	250 (100.0)	164 (100.0)	414 (100.0)	

Discussed contraception with health care provider, current pregnancy	No Yes	236 (73.07) 87 (36.93)	108 (60.67) 70 (39.33)	344 (68.66) 157 (31.34)	0.004
	
	Total	323 (100.0)	178 (100.0)	501 (100.0)	

Current pregnancy was planned	No Yes	142 (43.96) 181 (56.04)	108 (60.67) 70 (39.33)	250 (49.9) 251 (50.1)	0.000
	
	Total	323 (100.0)	178 (100.0)	501 (100.0)	

Timing of disclosure	Immediately Within 1 year >1 year No response	207 (63.7) 24 (7.4) 19 (5.8) 75 (23.1)	99 (55.6) 19 (10.7) 7 (3.9) 53 (29.7)	306 (60.8) 43 (8.5) 26 (5.2) 128 (25.5)	0.000
	
	Total	325 (100.0)	178 (100.0)	503 (100.0)	

Reasons for disclosure	Partner health Own health Child health Family support Other	134 (53.2) 41 (16.3) 8 (3.2) 12 (4.8) 57 (22.6)	68 (54.0) 26 (20.6) 4 (3.2) 14 (11.1) 14 (11.1)	202 (53.4) 67 (17.7) 12 (3.2) 26 (6.9) 71 (18.8)	0.019
	
	Total	252 (100.0)	126 (100.0)	378 (100.0)	

Awareness of partner’s status	Negative Positive Don't know	74 (27.1) 110 (40.3) 89 (32.6)	27 (17.9) 52 (34.4) 72 (47.7)	101 (23.8) 162 (38.2) 161 (38.0)	0.006
	
	Total	273 (100.0)	151 (100.0)	424 (100.0)	

Tested for TB, current pregnancy	No Yes	286 (88.5) 37 (11.5)	163 (91.6) 15 (8.4)	449 (89.6) 52 (10.4)	0.288
	
	Total	323 (100.0)	178 (100.0)	501 (100.0)	

Tested for TB, previous pregnancy	No Yes	246 (89.8) 28 (10.2)	137 (87.8) 19 (12.2)	383 (89.1) 47 (10.9)	0.531
	
	Total	274 (100.0)	156 (100.0)	430 (100.0)	

TB discussed, current pregnancy	No Yes	274 (84.6) 50 (15.4)	144 (80.9) 34 (19.1)	418 (83.3) 84 (16.7)	0.292
	
	Total	324 (100.0)	178 (100.0)	502 (100.0)	

There were significant differences in gestational age at first visit between groups at the two health facilities, as the majority of women at BDHC attended before 28 weeks (58.4% presented between 21 and 28 weeks) while 40.6% of women accessing care at PMH only presented after 28 weeks. Only 4% of women accessing services at BDHC attended four or more ANC visits, compared to 15.3% at PMH.

### Counselling and testing

The majority of women participating in our study reported receiving PMTCT counselling during their first ANC visit (86%, table 3) -with no difference across sites- while 19.8% received sdNVP during this visit. Less than a third of women (31.34%) had been counselled on contraception during ANC visits for this pregnancy, and only half reported the pregnancy as a planned pregnancy.

Disclosure rates were relatively high among the women in our sample, with almost three quarters responding that they had disclosed to their partners and 60.8% reportedly doing so immediately following diagnosis (Table 3). The main reasons provided for disclosure were to protect the health of their partners (53.4%) and themselves (17.7%). However, almost 40% of women did not know their partner’s HIV status; women from BDHC were less likely to know their partner’s status.

TB testing was rare in with only 10% of women being tested during this pregnancy or a previous pregnancy (Table 3): 10.4% were tested during this pregnancy, 10.9% during a previous pregnancy, with no difference between sites. Testing was discussed with 16.7% of the women, with slightly more women participating in discussions at BDHC than PMH (15.4% and 19.1%, respectively).

## Discussion

There is evidence that Kenya has made progress in PMTCT as HIV incidence among children between six weeks and one year is estimated to have declined from 27% to 10-15% within five years [3]. Our results show that, in our study population, there is high coverage for some recommendations, such as PMTCT counselling (86%) or provision of nevirapine to newborns: preliminary analysis of follow up data in our study (not presented here) suggests that 91.9% of newborns received sdNVP after birth, compared to 71% of mothers. However, implementation of other recommendations still faces challenges, especially in the context of the 2012 PMTCT guidelines and there focus on earlier treatment with Option B.

PMH is a major maternity hospitals in Nairobi and considered a “model institution for the PMTCT programme in Kenya”, one of the first institutions to offer PMTCT services based on national guidelines [4]. As such, PMH receives many referrals from surrounding health centres which could account for the greater client diversity and the higher number of women attending four or more ANC visits - as these may be higher risk pregnancies. It is also plausible that women may want to deliver at this facility but receive their antenatal care elsewhere. This could contribute to the higher number of women whose first ANC visit at PMH was at a later gestational age, as they register only in time for delivery. This trend is also evident in the higher number of women presenting at PMH that could not be enrolled in the study as they were past the cut off (36 weeks). At BDHC, a community health centre, only five women were excluded for this reason, as opposed to 62 at PMH. The persistence of implementation challenges across our study sites, despite the significant differences in type of facility and clients served, suggest that the challenges identified are not facility-specific but are indicative of broader, systemic issues. Although we cannot generalize from our sample, we could speculate that these systemic issues could also pose barriers to the effective implementation of PMTCT guidelines elsewhere and could potentially be exacerbated by Kenya’s recent move to free maternal health care, which could increase patient load [5].

None of the women in our sample presented for ANC prior to 20 weeks gestation. Although presenting to an ANC clinic at 20 weeks gestation fits with previous PMTCT guidelines, it poses a challenge to the earlier initiation of ART at 14 weeks proposed in the 2012 guidelines. Earlier initiation will only be feasible if women and health care providers are made aware of the importance of earlier ANC visits and women have access to antenatal care before 20 weeks gestation. The high percentage of women in our sample who attended four or less ANC visits also suggests that monitoring women once they are initiated on treatment may be complicated, more so since Efavirenz is indicated as part of the first line ARV regimen for women with no exposure to nevirapine, or where the exposure occurred more than 12 months prior. Though the guidelines allows for the substitution of nevirapine for Efavirenz during the first trimester, Palombi and colleagues (2011) have criticized the inclusion of Efavirenz in contexts that may provide limited monitoring due to the lack of knowledge of its effects on later stages of pregnancy, the potential for lifelong Efavirenz use, and the risk of hepatic toxicity, amongst others [6]. Hepatic toxicity is also a concern with nevirapine and would still require follow up. Providing adequate monitoring of treatment will be complicated if women attend less than four ANC visits or if women access care at multiple facilities and there is no continuity of care – preliminary analysis of follow up data from our study (not presented here) suggest that many women access ANC at multiple sites. The PMTCT guidelines state that women should be given sdNVP (for themselves and their infants) during “first contact” [3]. This is especially important in Kenya where a high percentage of women deliver outside of healthcare facilities. Yet, less than 20% of women in our sample, regardless of site, reported receiving the nevirapine; this is lower than what has been previously reported in Kenyan context [7]. Although Nairobi province has the lowest prevalence of home deliveries in Kenya, with an estimated 9.9% of deliveries taking place at home, it is possible the rates may differ among groups in the city since increased number of home deliveries tend to be associated with lower education and fewer ANC visits [9]. Therefore, the potential for transmission remains, especially considering that not all women may take sdNVP, even if they have it in their possession [2]. It is also possible that the nevirapine was given at later ANC visits, as has been reported elsewhere in Kenya [2] – this seems to be supported by preliminary analysis of our follow up data (not presented here) that suggest that 91.9% of newborns received sdNVP following birth and 71% of mothers received sdNVP at the onset of labour. While the high coverage at labour is encouraging, this still deviates from the guidelines that clearly state that sdNVP should be given to women at first contact.

According to the Essential Package of Integrated Antenatal Care Services outlined in Kenya’s PMTCT guidelines, women should receive counselling in accordance with positive prevention principles and be tested for opportunistic infections such as TB during ANC. Contraception information and encouragement to disclose are two elements of positive prevention included in counselling. Very few women in our study had discussed contraception with a health care provider, although this may be due to the fact that the data used for this analysis was collected during enrolment, at which time women may only have attended their first ANC visit. It is highly likely that family planning and contraception would only be discussed at later visits, or post-partum [9], and we will explore this in analysis of our follow up data. However, the high percentage of women who reported their current pregnancies as unplanned seem to support the idea that access to contraception is still limited for HIV-positive women in Kenya, and the lack of discussion of family planning during ANC visits presents a missed opportunity. It also contradicts the importance accorded to reducing unplanned pregnancies among HIV positive women in the guidelines – the second prong of the adopted four-prong approach to PMTCT. Barriers to accessing contraception including non-disclosure, lack of staff with adequate training in family planning counselling or lack of time, ideology, and gender inequity will need to be addressed for the guidelines to effectively reduce PMTCT [1,10].

Although more than two thirds of the women in our sample had disclosed their status to their partners, this data was collected in the context of a study that sent text messages about PMTCT to participants and where disclosure had initially been an inclusion criteria. Given that the majority of women in our study used phones that belonged to either their partners or family members, it is plausible that many women who had not disclosed may have declined participation for fear of involuntary disclosure. Women’s lack of knowledge about their partner’s status further indicates that discussions about HIV status and disclosure is still a challenge. This lack of discussion decreases the potential for men to be involved in PMTCT, increasing challenges to many aspects of the PMTCT guidelines, including the use of condoms [1].

The guidelines state that all pregnant women who are HIV positive should be screened for TB. However, our data suggests that TB testing is rare, both at PMH and BDHC, suggesting that this is also a systemic issue that may need to be addressed at a higher level. Given the high rates of co-infection in Africa, guidelines have also suggested that TB and HIV services be integrated, but studies from Cameroon and South Africa have discussed the barriers to this posed by local infrastructure and human resource availability [11]. Similar barriers may need to be addressed in the context of PMTCT, specifically in the context of free maternal health and the resultant increase in patient load.

### Limitations

The data presented here was collected in the context of a RCT examining the role of mHealth in PMTCT. The focus on pregnant women accessing PMTCT services limited our sample to women who are HIV positive and we cannot compare access to ANC with that of HIV negative pregnant women. Given that the data was collected in the context of a RCT, the sample was not randomly selected so our findings are only applicable to the population studied and cannot be generalized to the population of HIV-positive women attending ANC in Kenya. The generalizability of our findings may also be affected by the study setting: the study was conducted at only two facilities in urban areas and PMH is a major health centre. It is more plausible that these centres would have the infrastructure needed to adhere to the guidelines than those in rural areas. Caution is warranted in extrapolating results to HIV-positive women in Kenya generally.

The primary purpose of our study was to examine the role of mHealth in PMTCT and not to examine the fidelity of guideline implementation. As a result, some indicators of guideline implementation, such as HIV testing or the obstetric management and care received during delivery, included in other studies may have been excluded (see for instance Musalia et al., 2010 [4]). Similarly, drawing blood samples for CD4 counts was part of our study protocol and could not be assessed as an element of the guidelines. Another limitation is our reliance on preliminary analysis of the data collected at enrolment and excluding data from follow-up questionnaires (not yet completed) which limited the scope of the data we could present to illustrate challenges to implementation.

## Conclusions

The WHO’s 2010 revised PMTCT guidelines moved towards reducing the gaps between standard of care for PMTCT in high and low resource settings. This has led to renewed optimism for elimination of mother-to-child transmission and meeting the targets set out by the Joint United Nations Programme on HIV/AIDS. However, guideline implementation continues to face challenges such as late presentation for care after 20 weeks gestation, less than four ANC visits, poor screening for opportunistic infections such as TB, poor contraception coverage, and low disclosure rates. Effective scale up and implementation of PMTCT services requires that such ongoing program challenges be identified and appropriately addressed, with consideration of the local context. The greatest ongoing challenge to PMTCT is not knowing what needs to be done, but ensuring that what needs to be done is implemented.

## List of abbreviations

ANC: Antenatal care; ART: Antiretroviral therapy; ARV: Antiretroviral; BDHC: Baba Dogo Health Centre; DFATD: Foreign Affairs, Trade and Development Canada; GHRI: Global Health Research Centre; HAART: Highly active antiretroviral therapy; IDRC: International Development Research Centre; PMH: Pumwani Maternity Hospital; PMTCT: Prevention of mother-to-child transmission of HIV; RCT: Randomized control trial; sdNVP: single dose Nevirapine; SSA: sub-Saharan Africa; TB: Tuberculosis

## Competing interests

The authors declare no competing interests.

## Authors' contributions

EdP drafted the manuscript; SYS analyzed all data. MG was involved with the implementation and coordination of the study. LG, BBE, RL, and JK were involved in the conception and implementation of the study; LSA conceived of the manuscript and reviewed the final draft.

## Declarations

The publication costs associated with this article are funded by Foreign Affairs, Trade and Development Canada and the International Development Research Centre through the Global Health Research Initiative.

This article has been published as part of *BMC Health Services Research* Volume 14 Supplement 1, 2014: Uptake and impact of research for evidence-based practice: lessons from the Africa Health Systems Initiative's research component. The full contents of the supplement are available online at http://www.biomedcentral.com/bmchealthservres/supplements/14/S1
